# Individual Differences in Aesthetic Preferences for Multi-Sensorial Stimulation

**DOI:** 10.3390/vision4010006

**Published:** 2020-01-06

**Authors:** Jie Gao, Alessandro Soranzo

**Affiliations:** 1 Institute of Education, University College London, London WC1H 0AL, UK; 2 Department of Psychology, Sheffield Hallam University, Sheffield S10 2BP, UK; A.Soranzo@shu.ac.uk

**Keywords:** multi-sensorial stimulation, individual difference, aesthetic preference

## Abstract

The aim of the current project was to investigate aesthetics in multi-sensorial stimulation and to explore individual differences in the process. We measured the aesthetics of interactive objects (IOs) which are three-dimensional objects with electronic components that exhibit an autonomous behaviour when handled, e.g., vibrating, playing a sound, or lighting-up. The Q-sorting procedure of Q-methodology was applied. Data were analysed by following the Qmulti protocol. The results suggested that overall participants preferred IOs that (i) vibrate, (ii) have rough surface texture, and (iii) are round. No particular preference emerged about the size of the IOs. When making an aesthetic judgment, participants paid more attention to the behaviour variable of the IOs than the size, contour or surface texture. In addition, three clusters of participants were identified, suggesting that individual differences existed in the aesthetics of IOs. Without proper consideration of potential individual differences, aesthetic scholars may face the risk of having significant effects masked by individual differences. Only by paying attention to this issue can more meaningful findings be generated to contribute to the field of aesthetics.

## 1. Introduction

Aesthetics play an important role in everyday life. We buy a mug because we admire its attractive design; we choose a particular hotel room because we like the view or the decoration. Such aesthetic experiences are rooted in our brain; yet, there is little scientific understanding of how we make these aesthetic decisions.

Research in experimental aesthetics faces different challenges, the two main ones being that: (i) Aesthetics often arise from multi-sensorial stimulation; and (ii) aesthetic experience is essentially subjective.

### 1.1. Aesthetics in Multi-Sensorial Stimulation

**Figure 1:**
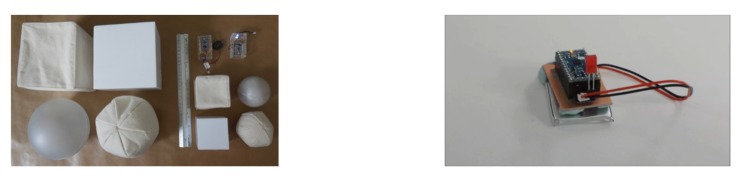
Picture of interactive objects (IOs) and the motion sensor.

In brand design, much attention is paid to a consumer’s whole experience rather than to a single product feature [1,2]. The design builds upon the evidence that when multiple senses are stimulated simultaneously, it leads to a richer and more immersive experience [3]. However, aesthetic psychologists have been studying each sense in isolation and mainly focusing on the visual sense. Just as Carbon and Janesch [4] pointed out, whilst vision is the sense that has been widely studied in aesthetics, in many circumstances haptic and tactile features may overpower visual features in terms of pleasure. They have, therefore, argued that any model which describes aesthetic responses must consider more than one sense at a time (see also Muth et al. [5]). To explore how different senses contribute to the overall aesthetic experience, Soranzo et al. [6] studied the aesthetic preference for Interactive Objects (IOs). As shown in Figure 1, these are three-dimensional objects, which contain electronic components that exhibit an autonomous behaviour when handled, e.g., vibrating, playing a sound, or lighting-up. (It will be interesting, in the future, to manipulate additional dimensions, such as the IO’s colour or their odour.) IOs are an ideal device to investigate aesthetics as they stimulate more than one sense at a time. By employing the IOs which differ in size, surface texture, contour and behaviour (as illustrated in Table 1), the current project aimed to investigate individuals’ aesthetics in multi-sensorial stimulation.

### 1.2. Individual Differences in Aesthetics

The Latin adage “de gustibus non est disputandum” (there is no accounting for taste) suggests that individual differences in aesthetic are either arbitrary or otherwise inexplicable [7,8]. However, modern behavioural research has shown that meaningful statements can be made about individual differences in aesthetics [9], which actually generates more insights into the study of aesthetics. Appelt et al. [10] underlined that in situations where no clear tendency emerges from the overall sample, it is not unlikely that individual differences have cancelled out the expected effect and that the effect is evident, but only for a subset of participants. This is why psychologists may want to explore individual differences even when this is not their primary goal. By investigating individual differences in aesthetics, scholars found stable and statistically robust individual preferences which were masked by weak population preference [11,12].

While many studies have found a consistent aesthetic preference for stimuli with specific properties (e.g., simplicity in vision [13]; smoothness in touch [14]), scholars are reluctant to use these findings to claim the universal nature of these aesthetic appeals. This is because individual differences have emerged in practically all studies. As long as a century ago, Thorndike [15] pointed out that the diversity among people’s preference is vast and that “*although any one person may feel very decided preferences, these are never shared by enough of his fellows to make anything like universal agreement*” (p. 150). Aesthetic response is an intrinsically subjective and whimsical experience. There are probably no other scientific field where individual differences are more relevant. Modern behavioural research on empirical aesthetics has shown that scientifically meaningful statements of individual difference can be made. For example, McManus et al. [12] found that people can be categorised into two clusters based on their preferences for rectangles: One cluster preferred rectangles closer to a square shape, and the other cluster preferred elongated rectangles; Spehar, Walker and Taylor [16] identified two clusters of participants based on their appreciation of fractal patterns: One cluster liked images with extreme values of the spectrum slope, and the other cluster preferred intermediate slope values. These individual differences are worth further exploring. It could be argued that an aesthetic appeal may only be shared within distinct clusters of individuals, rather than universally.

### 1.3. The Qmulti Protocol

In order to incorporate the exploration of individual difference into the investigation of overall aesthetic experiences, Gao and Soranzo [17] developed the Qmulti protocol which is based on Q-methodology (see References [18,19] for more details). The key aspects of the Qmulti protocol are presented as the following, namely, the Q-sorting procedure and corresponding Q-factor analysis; the distinction between preference and dominance; and the dedicated R script of Qmulti protocol.

#### 1.3.1. The Q-Sorting Procedure and Corresponding Q-Factor Analysis 

**Figure 2:**
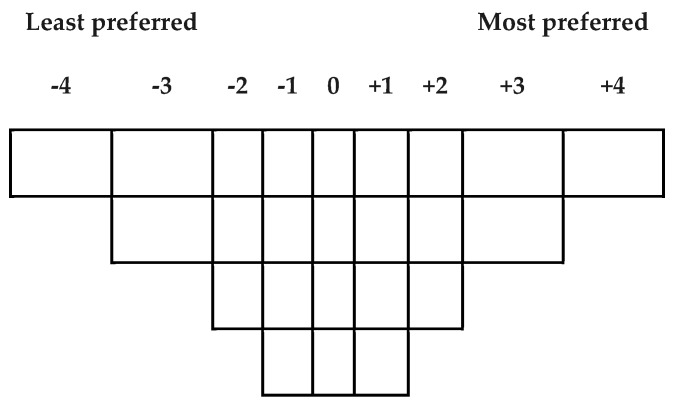
A q-sorting grid for an experiment with 24 stimuli (or items).

The Q-sorting procedure proposed by Stephenson [18] requires participants to rank-order stimuli (e.g., statements, pictures, objects, etc.) into one single quasi-normal (i.e., bell-shaped) response grid which represents a continuum of preference, or agreement, or importance, etc. An example of the Q-sorting grid is presented in Figure 2. The shape of the grid depends on the research questions. Watts and Stenner [19] have provided effective instructions on how to build the response grid. While a quasi-normal distribution shape is commonly used in Q-sorting, researchers should design the grid based on their knowledge of the research topic under investigation [19].

Q-factor analysis [18] is used to analyse the Q-sorting data. In contrast to conventional factor analysis, Q-factor analysis groups participants together instead of items. It enables researchers to identify consensus and disagreement among participants. Each Q-factor represents the shared aesthetic judgment among the participants who are significantly loaded on the Q-factor. In this way, individual differences can be explored and informed by the data.

After Q-sorting, participants are asked to clarify the reasons for their aesthetic judgments, such as why they prefer one stimuli over the other; which stimuli feature(s) draws their attention, etc. The qualitative data complements the Q-sorting data to give an in-depth and comprehensive account of how different clusters of participants make aesthetic judgments in a multi-sensorial stimulation setting.

#### 1.3.2. The Distinction between Preference and Dominance

As mentioned earlier, people pay attention to a variety of information when making aesthetics decisions. For example, when judging the aesthetics of a coloured polygon, people may consider variables, such as the shape or the colour or the combination. People may differ in their preferences within a certain variable, but agree on the importance of the variable. For example, individual A may prefer red polygons whilst individual B may prefer blue polygons; but both A and B may regard the colour as the most important variable on which they base their aesthetic judgment. We refer to the importance of a variable as its dominance. The analysis of dominance has a similar meaning in a regression analysis of finding out whether a variable can predict an outcome, but it utilises the Q-sorting data directly and provides a more straightforward means of addressing this issue [16]. Mathematically, the dominance of a variable is a measure of the spread of its levels across the Q-sorting grid: The larger the spread (i.e., extreme positions in the grid, such as very much liked and very much disliked), the higher the weight.

#### 1.3.3. The Dedicated R Script of Qmulti Protocol

A ready to use R script [20], the QmultiProtocol.R, has been developed to analyse the data collected by the Q-sorting procedure. The QmultiProtocol.R makes use of the following packages: ‘qmethod’ [21]; ‘ordinal’ [22]; and ‘data.table’ [23]. The default version of the QmultiProtocol.R adopts the frequentist approach and runs the ordered-probit model to analyse the Q-sorting data. It uses the ordered-probit model to analyse the preferences whilst the dominance is tested via the analysis of variance of the weight of each variable, calculated by measuring the spread across the grid of the levels of each variable (see Reference [17] for more details). 

### 1.4. Current Project

The aim of the current project was to investigate aesthetics in multi-sensorial stimulation and to explore individual differences in the process. To achieve this aim, we measured the aesthetics of IOs using the Q-sorting procedure. Data were analysed by applying the QmultiProtocol.R [17]. By following the Qmulti protocol, we were able to address the following research questions:

- Which are the overall preferred characteristics of each variable of the IOs?
- Which are the important variables that influence people’s preference of the IOs?
- Do people systematically differ in their aesthetic judgement about the IOs?
- Do different clusters of people prefer different characteristics of a variable of the IOs?
- Are different clusters of people driven by different variables of the IOs?

## 2. Method

### 2.1. Participants

Eighteen participants (14 females and 4 males, aged 18–24 years old) took part in the Q-sorting experiment of IOs.

### 2.2. Materials

Four variables of the IOs were manipulated, namely, Size, Surface texture, Contour and Behaviour (see Figure 1). The variables differ in the number of levels, as indicate in Table 1. As a result, there are 32 IOs in total.

### 2.3. Procedure

**Figure 3:**
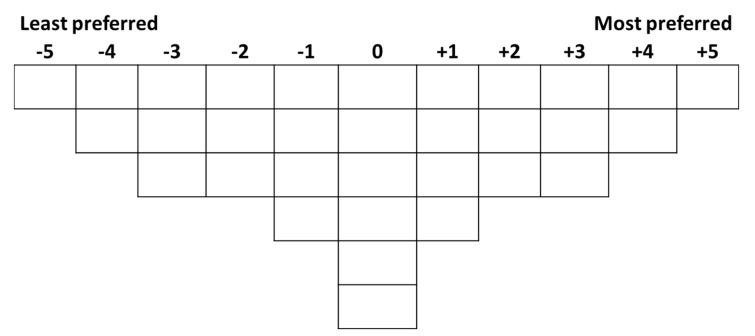
The response grid of Q-sorting procedure.

**Figure 4:**
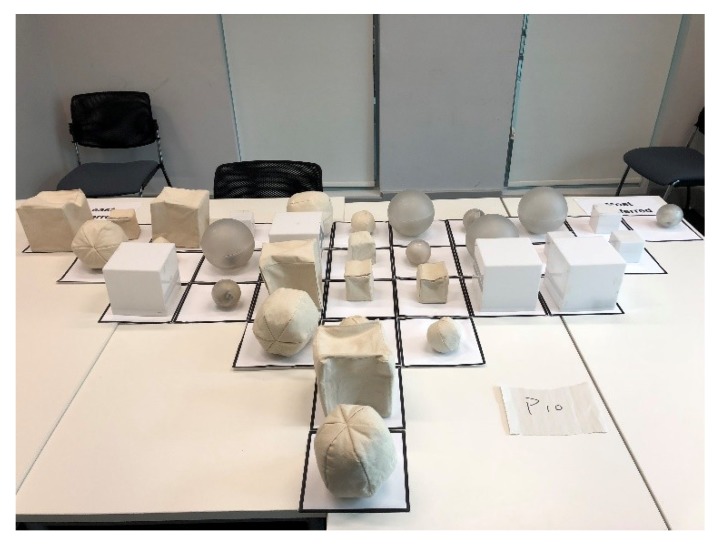
Example of Q-sorting result.

Participants were first asked to play with the 32 IOs to familiarise themselves with the objects, especially the behaviour that each IO exerts when picked up. Then participants were asked to rank-order all the IOs into one single bell-shaped (i.e., quasi-normal) grid ranging from ‘the least preferred’ (−5) to ‘the most preferred’ (+5) (see Figure 3 and Figure 4). IOs that were sorted into one common column were considered as equivalent. The grid was designed to (a) accommodate the 32 IOs and (b) enable participants to differentiate their preferences of the IOs. After Q-sorting, participants were asked to elaborate on their aesthetic judgement.

### 2.4. Ethics

All participants gave their informed consent for inclusion before participation in the experiment. The study was conducted in accordance with the Declaration of Helsinki. Ethical approval was obtained from the Ethics Committee of Sheffield Hallam University (Ref: ER6377599).

## 3. Results

The Q-sorting data were inserted into a spreadsheet to be read by the QmultiProtocol.R in R. Detailed steps of the analysis performed by QmultiProtocol.R can be found in Reference [17]. An example of data collected from a participant (P10) is shown in Figure 4. The results of the analysis are presented in the following sections.

### 3.1. Research Question One: Overall Preference

**Figure 5:**
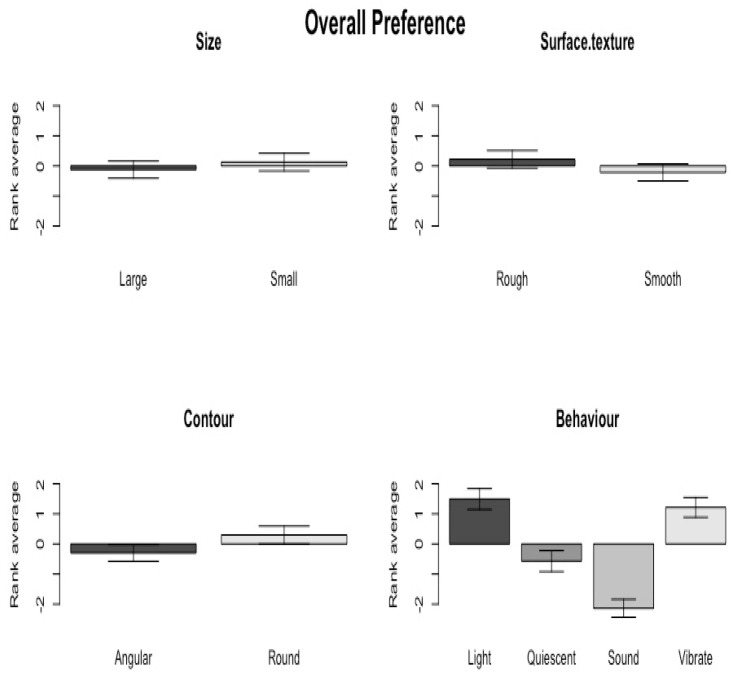
The overall preference of each variable.

To find out the overall preferred characteristics of each variable of the IOs, the QmultiProtocol.R runs an ordered-probit model on the ranks that each of the IOs received by all the participants. Specifically, the result of Wald chi-square test suggested that in general, participants preferred rough to smooth surface texture (*Χ*^2^ = 6.44, *df* = 1, *p* = 0.004), round to angular shape (*Χ*^2^ = 10.38, *df* = 1, *p* = 0.001) and lighting/vibrating to sounding/quiescent objects (*Χ*^2^ = 206.74, *df* = 3, *p* < 0.001). However, there was no significant difference in preference between big and small objects (*Χ*^2^ = 2.05, *df* = 1, *p* = 0.15). Figure 5 illustrates the overall preference of each variable.

### 3.2. Research Question Two: Overall Dominance

**Figure 6:**
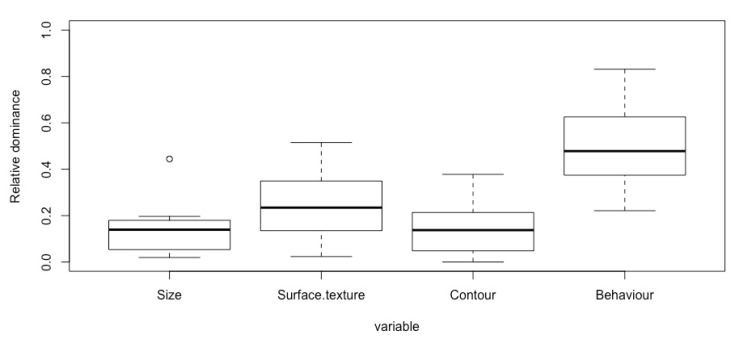
The overall dominance of each variable.

To find out which are the important variables that influence people’s preference of the IOs, the QmultiProtocol.R conducts an analysis of variance on the weights of variables that are calculated by the analysis procedure (see Reference [17] for more details). The result suggested that there was a significant difference between the variables in terms of how important they were considered by the participants when making an aesthetic judgment (*F* (3, 68) = 28.34, *p* < 0.001). Among the four variables, the Behaviour variable had the highest dominance weights, suggesting that participants paid more attention to the behaviour than the size, surface texture and contour of the IOs. Figure 6 illustrates the overall dominance of each variable.

### 3.3. Research Question Three: Individual Differences

Q factor analysis was conducted with the Q-sorting data (see Reference [24] for a detailed Q factor analysis procedure). We compared different factor solutions (i.e., two Q-factors, three Q-factors and four Q-factors) based on the variance that the factors account for, the Eigenvalue and the meaningfulness of the factor scores. As a result, a three-factor solution was chosen, which accounted for 70% of the variance in total. Eight participants were significantly loaded on Q-Factor1 (Eigenvalue = 5.4, variance= 30%), six on Q-Factor 2 (Eigenvalue = 4.1, variance = 23%) and three on Q-Factor 3 (Eigenvalue = 3.1, variance = 17%). This suggested that there were three clusters of participants who made a different aesthetic judgment of the IOs.

**Figure 7:**
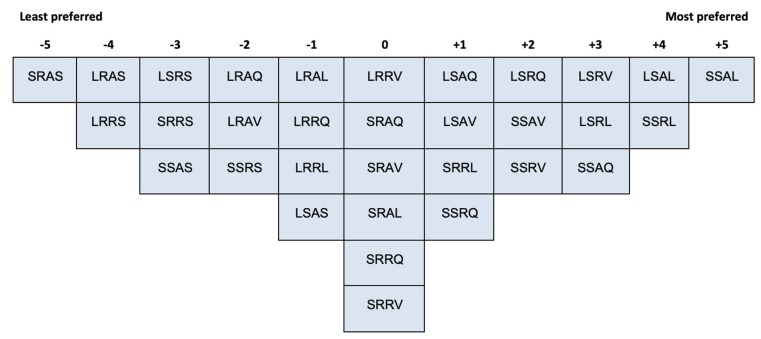
The shared Q-sorting result of participants loaded on Q-factor 1.

**Figure 8:**
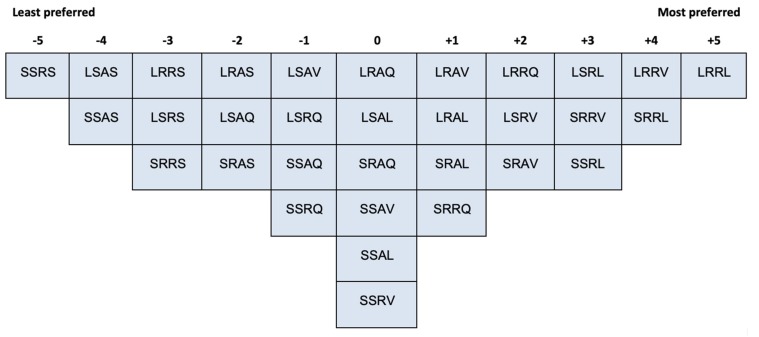
The shared Q-sorting result of participants loaded on Q-factor 2.

**Figure 9:**
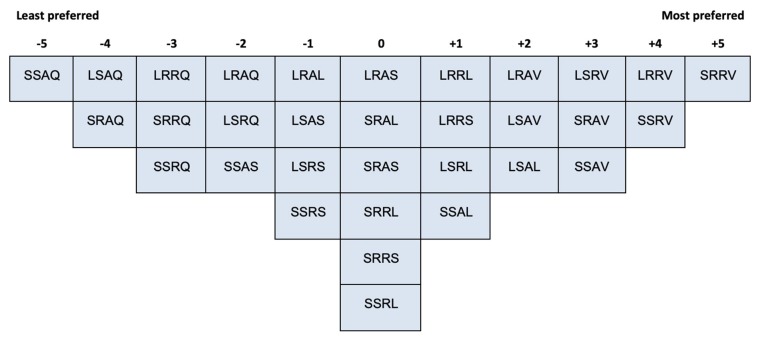
The shared Q-sorting result of participants loaded on Q-factor 3.

Figure 7, Figure 8 and Figure 9 illustrate the shared aesthetic judgments among the participants who were significantly loaded on each Q-factor. The first letter in each cell represents the IO’s size (L = Large, S = Small); the second is the surface texture (R = Rough, S = Smooth); the third is the contour (A = Angular, R = Round); and the forth is the behaviour (Q = Quiescent, V = Vibrate, L = Light, S = Sound). The following paragraphs elaborate on the patterns of the shared aesthetic judgment, which help readers to read the figures.

As can be seen, participants loaded on Q-factor 1 ranked the small smooth angular object which lights up as the most preferred one (+5) and the small rough, angular object which makes a sound like the least preferred one (−5). By examining the pattern of the ranks of IOs, we can see that participants of Q-factor 1 tended to prefer smooth surface to rough surface, and they dislike the IOs that make a sound. To be more specific, the three most preferred IOs are all smooth lighting up objects, whereas, the three least preferred objects are all rough sounding objects. No clear pattern is identified for the size or the contour variable. The qualitative data of post-sorting interview provide further evidence to support this shared aesthetic judgment. For example, participant P10 reported, “*I prefer the smooth than the fabric texture because it feels clean, smooth and easy to clean stuff like that; I don’t really have a preference of the circles or the squares, but I like lighting up*”.

On the other hand, participants loaded on Q-factor 2 preferred rough round IOs, just like participant P09 pointed out “*[the fabric balls] feel comfortable to hold*”. Meanwhile, they seemed to prefer vibrating and lighting-up IOs to sounding IOs. For example, participant P03 said in the post-sorting interview “*I found the sound ones are quite harsh*”. Similar to Q-factor 1, no clear pattern for the size variable is identified. 

As for participants loaded on Q-factor 3, they ranked quiescent objects as the least preferred because “*they are just boring*” (P11). They liked vibrating IOs the most, just as participant P12 indicated “*I prefer vibration to everything else. It’s like massage*”. While participants loaded on Q-factor 1 and 2 ranked sounding IOs as the least preferred, participants loaded on Q-factor 3 preferred sounding IOs to quiescent ones. Participant P17 explained that “*I never heard a ball making a sound, it’s quite cool*”. 

**Figure 10:**
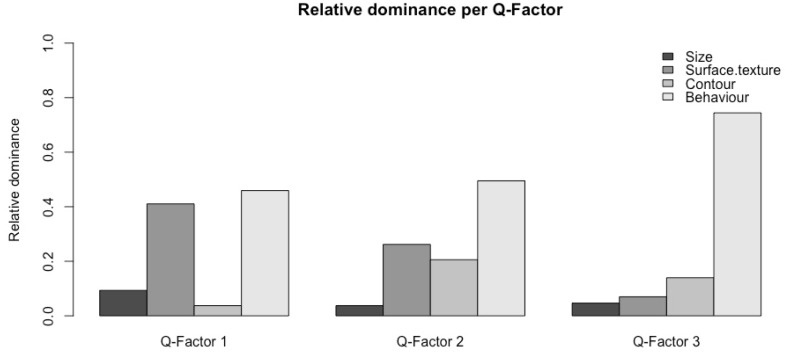
The dominance of variables for each Q-factor.

To further explore which variable(s) each cluster of participants paid more attention to, the dominance weights of variables were calculated separately for each cluster of participants (see Table 2). By inspecting these weights, we can infer that: Participants who were significantly loaded on Q-factor 1 mainly based their aesthetic judgement on the behaviour and the surface texture; participants loaded on Q-factor 2 mainly considered the behaviour, but still paid certain attention to the surface texture and contour; and participants loaded on Q-factor 3 mainly focused on the behaviour with little attention paid to the other variables. Figure 10 illustrates the dominance of variables for each factor.

### 3.4. Research Question Four: Interaction between Individual Differences and Preferences

**Figure 11:**
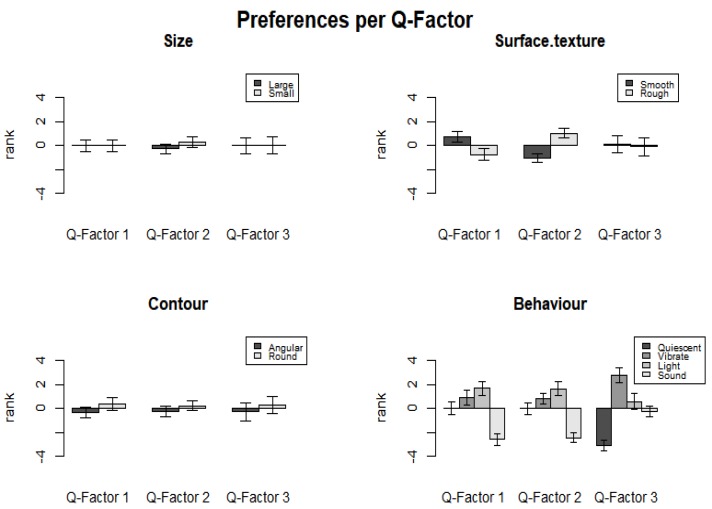
The preference of variables for each Q-factor.

The aim of this analysis is to find out whether participants of different clusters differ in their preference for IOs. Table 3 illustrates the results of Wald chi-square tests for the interaction between individual differences and preferences. As can be seen, there was no significant interaction among the four variables of the IOs when all Q-factors were considered together. In terms of selected comparison, Figure 11 shows that Q-factor 1 and Q-factor 2 mainly differed in their preferences of surface texture, that is, participants loaded on Q-factor 1 preferred smooth texture, whereas, participants loaded on Q-factor 2 preferred rough texture. Meanwhile, Q-factor 3 mainly differed from Q-factor 1 and 2 in respect of the behaviour variable, as shown in Figure 11.

### 3.5. Research Question Five: Interaction between Individual Differences and Dominance

The aim of this analysis is to find out whether participants of different clusters differ in the variable(s) they mostly consider when ranking the IOs. An analysis of variance was conducted on the dominance weights of each Q-factor.

**Figure 12:**
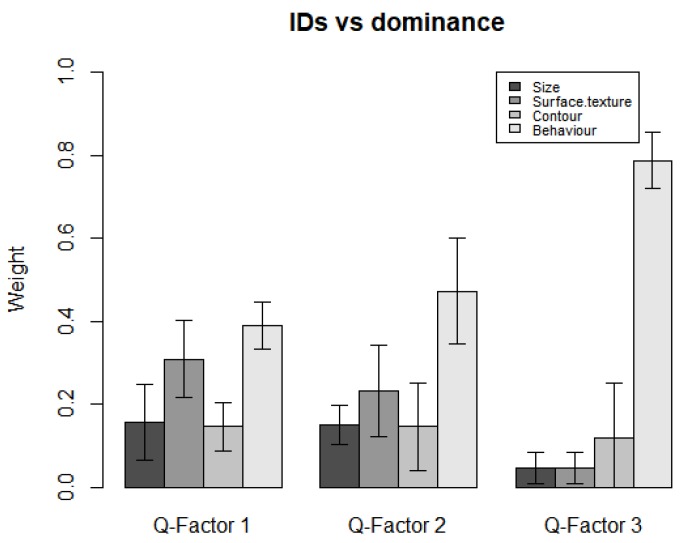
The interaction between individual differences and dominance.

The result of analysis of variance suggested that participants loaded on Factor 3 were significantly different from the participants loaded on Q-factor 1 and 2 in terms of the variables that they paid attention to when making an aesthetic judgment (*F* (6, 56) = 8.02, *p* < 0.001). Figure 12 illustrates the interaction between individual differences and dominance weights of the variables of the IOs. 

## 4. Discussion

In this project, we examined aesthetics in multi-sensorial stimulation and explored individual differences in the process. By adopting the Q-sorting procedure, we investigated participants’ aesthetics of Interactive Objects (IOs), which stimulate more than one sense at once. Data were analysed using the QmultiProtocol.R [17] which distinguishes between preference (i.e., the preferred characteristic of a stimulus) and dominance (i.e., the most important variable(s) in aesthetic judgment). By following the Qmulti protocol [17], it was possible to examine (i) the overall preferred characteristics of the IOs, (ii) the overall dominance, (iii) the individual differences; and the interactions between (iv) individual differences and preference and (v) individual differences and dominance. 

### 4.1. Overall Preferences

The analysis of overall preference revealed that participants preferred IOs that (i) vibrate, (ii) have rough surface texture, and (iii) are round. No particular preference emerged about the size of the IOs.

#### 4.1.1. Vibration

Among the variables studied in this project, “behaviour” was the dominant one (see detailed discussion below). Among the different behaviours of the IOs, the vibration was preferred by most of the participants. This result supports Carbon and Janesch’s [4] argument that haptic and tactile features enhance overall aesthetic experience.

#### 4.1.2. Roughness

In respect of the preference of surface texture, the findings of this study and previous research are controversial. On the one side, Ekman, Hosman and Lindstrom [14], and Etzi, Spence, and Gallace [25] found that smooth surfaces were preferred over rough ones; on the other side, Soranzo et al. [6]; Rowell and Ungar [26], and Jehoel, Ungar, Mccallum, and Rowell [27] reported the opposite findings. 

The degree of roughness might lead to a certain extent account for the inconsistent findings. In addition, psychophysics studies (e.g., Reference [28]) suggest that there exists an interaction between vibration and the perception of roughness. Therefore it can be argued that rough texture may be preferred over smoothness in multi-sensorial stimulation. However, the present study also found that one cluster of participants preferred smooth IOs whilst the other cluster preferred rough IOs. It is, therefore, possible that the discrepancies emerged in previous studies may be partially due to individual differences.

#### 4.1.3. Roundness

The result that participants preferred round IOs over squared ones is in line with previous literature (e.g., see the “smooth curvature effect”, [29]) which mainly focussed on 2D visual representations of static objects. The present study extended the findings, suggesting that this preference is very powerful and can be applied to 3D objects in multi-sensorial stimulation settings. 

#### 4.1.4. Size

Silvera, Josephs and Giesler [30] suggested that larger stimuli are usually preferred to smaller ones. This effect was not replicated in the present study. However, it should be noted that Silvera et al. [30] presented their stimuli pictorially rather than using physical objects. It is, therefore, possible that this preference is confined to the visual sense. Nonetheless, the lack of effect should be taken cautiously, considering the limited size range of our stimuli.

### 4.2. Overall Dominance

With regard to the dominance of the variables, we found that the most important variable considered by overall participants was the behaviour. This suggests that participants paid more attention to the behaviour than the size, surface texture or shape of the IOs. This is also supported by the qualitative data collected from the post-sorting interview. The behaviours exerted by the IOs were the most commonly mentioned reasons when participants were asked about the reasons, they preferred an IO to another.

This finding leads to the issue of aesthetic primitives. Latto [31] defined an aesthetic primitive as a primary or fundamental “stimulus or property of a stimulus that is intrinsically interesting…” (p. 68). Such aesthetic primitives, if they exist, may be hard-wired in the cognitive system, and may have an evolutionary basis. Although several stimuli features have been suggested to be primitives (e.g., golden ratio, symmetry, roundness, etc.); there is inconsistent evidence that they undoubtedly are primitives. Soranzo et al. [6] found that participants across various age groups, genders and cultural backgrounds preferred behaving objects over quiescent objects. Similar findings were found by the current study. Thus, we suggest that “behaviour” could be an aesthetic primitive.

### 4.3. Individual Differences

In addition to overall preference and dominance, we considered individual differences and the interaction between individual difference and preference and dominance, respectively. 

Q-factor analysis suggested that participants could be clustered into three Q-factors. Participants of Q-factor 1 preferred smooth Ios and disliked the Ios that make a sound. Participants of Q-factor 2 preferred rough round Ios and also disliked the sounding Ios. In contrast, participants of Q-factor 3 ranked the quiescent ones least preferred. By analysing the interactions between individual differences and preference, we found that participants loaded on Factor 1 preferred smooth surface texture, whereas, participants loaded on Factor 2 preferred rough surface texture. The analysis of the interaction between individual differences and dominance revealed that different clusters of participants laid emphasis on different variables when making an aesthetic judgement. Participants of Q-factor 1 and 2 pay attention to variables, such as surface texture and contour together with behaviour whilst participants of Q-factor 3 were mainly driven by the behaviour of IOs. These findings have highlighted the relevance of exploring and discussing individual differences in the field of aesthetics. Without proper consideration of potential individual differences, aesthetic scholars may face the risk of having significant effects masked by these individual differences. Only by paying attention to this issue can more meaningful findings be generated to contribute to the field of aesthetics.

## 5. Conclusions

By employing the Q-multi protocol, this research examined the aesthetics preference and dominance and the individual differences of multi-sensorial stimuli. The preferred characteristics of the stimuli were vibration, roughness and roundness. The dominance variable was behaviour, suggesting that “behaviour” could be an aesthetics primitive in Latto’s [31] terms. Findings have also highlighted the relevance of exploring and discussing individual differences in aesthetics.

## Figures and Tables

**Table 1:** Variables with corresponding levels.

Form			Behaviour
Size	Surface Texture	Contour	
Small (7.5 cm)	Smooth (plastic)	Round (sphere)	Emit a light
Large (15 cm)	Rough (fabric)	Angular (cube)	Play a sound
			Vibrate
			Quiescent

**Table 2:** Dominance weights of variables for each Q-factor.

	Q-Factor 1	Q-Factor 2	Q-Factor 3
**Size**	0.093	0.038	0.049
**Surface texture**	0.410	0.248	0.024
**Contour**	0.037	0.210	0.146
**Behaviour**	0.459	0.504	0.780

**Table 3:** Result of Wald chi-square test for the Preference per Q-factor interactions.

Variable	*df*	*Chi*-Square	*p*
Factor * Size	2	0.13	0.935
Factor * Texture	2	1.98	0.372
Factor * Contour	2	3.52	0.172
Factor * Behaviour	6	5.32	0.503

Note: * indicates interaction.
